# New proposed measures to evaluate contact tracing effectiveness: João Vasco Santos

**DOI:** 10.1093/eurpub/ckac131.215

**Published:** 2022-10-25

**Authors:** JV Santos, D Fernandes Silva, F Santos Martins, Espinho/Gaia Public Health Unit working group

**Affiliations:** 1 Public Health Unit, ACES Espinho/Gaia, ARS Norte, Porto, Portugal; 2 MEDCIDS, Faculty of Medicine, University of Porto, Porto, Portugal; 3 CINTESIS, Centre for Health Technology and Services Research, Porto, Portugal

## Abstract

**Issue/problem:**

Contact tracing is an important and widely used method to control transmission of communicable diseases. The COVID-19 pandemic presented a new and big challenge with a high number of confirmed cases and contacts and subsequent high burden on contact tracing activities. Naturally, the effectiveness of such interventions needs to be assessed, and appropriate measures are still rather underdeveloped.

**Description of the problem:**

We propose two new measures for assessing contact tracing effectiveness: “number needed to quarantine” (NNQ), which is the number of quarantined person-days needed to prevent one infectious person-day, and the proportion of infectious days prevented by the quarantined (PPID). We have applied these measures to assess COVID-19 contact tracing effectiveness in COVID-19 confirmed cases diagnosed between July and mid-September 2020 in a local Public Health Unity in the Northern region of Portugal (Espinho/Gaia). For robustness checks and accounting for the uncertainty of the infectiousness period, we used three different scenarios.

**Results:**

Depending on the infectiousness period considered, we have found a NNQ between 19.8 and 41.8 and a PPID between 19.7% and 38.2%. Contact tracing effectiveness was higher for some specific groups such as cohabitants and symptomatic contacts. Effectiveness also decreased with the increasing time from diagnosis or symptom onset to contact isolation

**Lessons:**

NNQ and PPID are straightforward and easy to use measures to evaluate contact tracing effectiveness in communicable diseases. Although this example focuses in the COVID-19 pandemic at a local setting, these measures can also be used for different communicable diseases and at different levels. This assessment step can be important for priority setting of transmission control activities but also on a health management perspective.

**Key messages:**

• New measures to evaluate contact tracing effectiveness are proposed: “number needed to quarantine” and “proportion of prevented infectious days”.

• These measures allow the identification of priority groups that must be quarantined, as well as time periods of intervention, for better transmission control.

